# A comprehensive transcriptomic and biochemical evaluation of a differentiation inducing anti-leukemic principle isolated from *Momordica charantia*

**DOI:** 10.1186/gb-2011-12-s1-p46

**Published:** 2011-09-19

**Authors:** Punit Prabha, Aparna Dixit

**Affiliations:** 1 School of Biotechnology, Jawaharlal Nehru University, New Delhi-110067, India

## Background

Acute myeloid leukaemia (AML) is a malignancy of the leukocytes or white blood cells. It represents a group of clonal haematopoietic stem cells having uncontrolled proliferation potential and at the same time defective for differentiation. Differentiation therapy is the most successful therapy till date available for the treatment of leukemia. Unlike chemotherapy and other therapeutic approaches, differentiation therapy mainly uses the cancerous cells to perform normal function by inducing them to differentiate and mature into terminally differentiated forms. All trans-retinoic acid (ATRA), Vitamin-D3 and Arsenic trioxide are the clinically proven therapies available for the treatment of AML. However, they are associated with several severe side effects. Therefore, novel alternate therapeutic drug development is an active area of investigation. Recent advances in genomics and proteomics has tremendous implication in drug development and understanding of its molecular mechanism in a reasonably short time period.

## Materials and methods

*Momordica charantia* seed extract was extracted using organic solvents and purified by TLC and NP-HPLC. The bioactive purified fraction was designated as P3 (Peak fraction 3). HL60 cells were treated with P3 and differentiation was estimated by NBT (Nitro Blue Tetrazolium) reduction assay and lineage of differentiation was confirmed by Wright-Giemsa staining and cell surface marker staining using by flow cytometry analysis. Signaling pathway was identified by kinase inhibitor treatment followed by P3 mediated induction of differentiation. Transcriptome profiling of the differentiating cells were performed by cRNA microarray with non-treated HL60 cells as a control and Affymatrix chips for hybridization of cRNA.

## Results

In the present study we have shown that P3 was able to differentiate HL60 cells into granulocytes by Wright-Giemsa staining, which was further confirmed by up regulation of CD15 and down regulation of CD14 by flow cytometry. Unlike other available therapeutic agents, P3 is not toxic even at 50 μg/ml concentration and resulted in ~56% of cell differentiation. Further, a detailed study of the change of expression of various genes involved in differentiation was done by c-RNA microarray. Microarray data suggested that 804 genes are up regulated, specifically in P3 treated cells, most of which are related to the metabolic pathway essential for differentiated cells and apoptosis. Moreover, 467 genes are down regulated and these down regulated genes are generally associated with cell cycle progression. Different kinase inhibitors were used to identify the signaling pathway involved in P3 mediated differentiation and we found that P3 follows MAPK pathway which was confirmed by PD98059 dependent inhibition of P3 activity. This result was supported by up regulation of MAPK associated genes in transcriptome analysis.

## Conclusion

Present study reports for the first time, a detailed study of an alternate drug, isolated from plant and its effect at signaling level as well as on the global gene expression of the differentiating leukemic cells. This study has potential implication for targeted drug designed for the treatment of Leukemia as well as other forms of cancer.

